# The Treatment of Uterine Fibroids

**Published:** 1907-04-27

**Authors:** H. T. Hicks

**Affiliations:** Assistant Surgeon to the Samaritan Hospital for Women; Obstetric Registrar to Guy's Hospital


### Gynæcology.

### THE TREATMENT OF UTERINE FIBROIDS.

By H. T. HICKS, F.R.C.S., Assistant Surgeon to the Samaritan Hospital for Women; Obstetric Registrar to Guy's Hospital.

### On some Points in the Treatment of Uterine Fibroids.

So many points must be taken into consideration in advising a patient suffering from uterine fibroids, that it is not always an easy matter to form a definite opinion. Therefore, it will perhaps not be amiss to criticise some of the points in the life-history and symptoms of these innocent but by no means harmless tumours.

### Influence of Age.

It is, of course, a well-known fact that fibroids become progressively more common after the age of 27 years, and that they grow steadily up to about the time of the menopause, when they share in the local atrophy, and shrink. Patients suffering from fibroids are told the above story and advised to await the menopause. If the menorrhagia is not profuse, drugs and other palliative measures are adopted in order to tide the patient over the menopause. In the greater number of cases this form of treatment should be adopted, but the prognosis is not always so simple. If a patient has reached 44 years and has one or more small interstitial fibroids, giving rise to moderate menorrhagia, she will no doubt reach the menopause with the aid of ergot and curetting. If, however, the menorrhagia is becoming more profuse, or the tumour is of large size, it surely is absurd to put off radical measures when chronic hæmorrhage and degeneration in the tumour will most likely play havoc with the patient in the meantime. One must be guided by the symptoms, the size and condition of the tumour and its anatomical position before deciding as to what advice to give. The age of the patient must certainly be taken into consideration, but it is a mistake to lead a patient to suppose that all danger will be passed when she reaches the menopause. Moreover, large fibroids most distinctly place the menopause later on in life, and menstruation may still be regular at the age of 52 or more.

### Influence of Menorrhagia and Bleeding on the Treatment.

Menorrhagia is perhaps the most common symptom which forces a woman to seek advice. Let us for a moment note what is happening in a uterus with a fibroid tumour. Fibroids start to grow in the uterine muscle, and as they steadily increase in size they tend to grow either towards the peritoneum (subperitoneal fibroid) or towards the uterine cavity (submucous). It is obvious that the closer the tumour is to the mucous membrane, the more pronounced will be the hæmorrhage, and *vice versâ.* With subperitoneal fibroids the hæmorrhage will become less and less until there may be no increase in the menstrual flow.

If an interstitial fibroid enlarges the cavity of the uterus, as it most commonly does, there will be an increase in the menstruating area. There will also be great increase in the vascularity, and thus menstruation becomes profuse. In time the tumour will project beneath the mucous membrane, or even become polypoid, when the mucous membrane is still more altered and continuous hæmorrhage may be the result. It is for hæmorrhage that we adopt palliative means of treatment. For pressure, pain, or other symptoms more radical methods must be used. We will, therefore, now discuss the various palliative methods by which the hæmorrhage may be controlled.

Of drugs which may do good ergot stands first on the list; it should be combined with iron (10-20 minims of the liquid extract with iron). It should be given just before and during the period, and will often control bleeding of moderate degree. The patient should rest during the period. I have seen quinine, hydrastis, and styptol do good, but no drug has so great an influence as ergot.

### Operative Palliative Treatment.

There are two chief forms of operative palliative treatment, *e.g.*, curetting and oöphorectomy. Curetting gives relief by removal of the œdematous and congested endometrium, but the relief is only temporary. If the hæmorrhage is very profuse, curetting should not be advised, because the fibroid is probably close beneath the mucous membrane, and it may be infected and become septic. Again, it is impossible satisfactorily to curette the mucous membrane of a greatly dilated uterine cavity. For large fibroids curetting is futile and should be avoided.

Curettage then will bring temporary relief for a few periods only, and should be used as a means of tiding the patient over the menopause; it should only be advised when the fibroid is interstitial, not of large size, and the uterine cavity only moderately distended. Oöphorectomy is another measure which has been tried as a palliative operative means of treating the menorrhagia of fibroids. When once the abdomen has been opened, it is, however, the tumour itself that should be removed; the ovaries, which have important physiological functions other than ovulation, should be conserved at all costs. There are other forms of palliative treatment which do not call for special comment. Oöphorectomy may bring on a most distressing menopause, and in many cases does not cause a cessation of growth in the tumour, and irregular uterine hæmorrhage continues.

Just a word about chronic uterine hæmorrhage. The more one sees of it the more convinced one becomes of its far-reaching effect on the mental and general condition of the patient. Chronic menorrhagia or metrorrhagia will wreck a woman's life, and a patient should be warned of the danger she has to face in the end. Fibroids are not the only innocent local cause of chronic hæmorrhage, for myometritis and arterial degeneration frequently cause it. Fibroids, however, are a very common cause. How often a patient who is the subject of chronic uterine hæmorrhage puts off going to her doctor, and how often does the doctor persist in palliative treatment in the hope that the menopause may bring the desired relief. A woman can stand a fairly prolonged period of bleeding, but a year to 18 months of moderate hæmorrhage is enough, and if the hæmorrhage is profuse, it may be too much. What is the result? A thin, nervous, lemon-yellow coloured woman comes for advice, having suffered, it may be, for years from menorrhagia. At home she is utterly incapable of doing any work, and her mental state may be bordering on delusional insanity. If a radical operation is carried out at once the shock may be sufficient to determine the threatened mental storm. The patient should be put to bed, fed up on good food and iron until she is in a condition to stand the radical operation. The mental side of these cases is, to my mind, often neglected.

### Pressure Symptoms and Pain.

These symptoms are most often associated with large tumours, but even small tumours may retrovert the uterus and become impacted in the pelvis. A sudden jerk may thrust a small fibroid backwards, as when a horsewoman puts her horse over a fence; the tumour may then become impacted in the pelvic cavity, causing great pain and pressure symptoms. Small tumours may be pushed out of the pelvis manually, and the symptoms thus relieved, but as a general rule a fibroid should be removed by operation if there are marked pressure symptoms and pain.

*The Size of the Tumour.*—A tumour that is larger than a three months' pregnancy should always be treated by radical means. It will sooner or later cause pressure symptoms, and is very prone to degenerate. Degeneration is more common about the menopause, and it must not be forgotten that a sarcoma associated with a fibroid occurs more frequently than can be explained by mere accident.

Fibroids grow slowly, and if the tumour begins to increase rapidly some form of necrosis is taking place within it, or sarcomatous changes are occurring. These changes are more common in the large fibroids than in the small tumours, and are particularly prone to occur when rapid vascular alterations are affecting the tumour such as are associated with the menopause and the puerperium, so that the very vascular changes at the menopause, which we look forward to as the cause of atrophy, may bring about one of the various forms of necrosis in the tumour.

### Anatomical Position.

*Subperitoneal Fibroids* do not give rise to much trouble unless they are of large size. As a rule a fibroid becomes fibrous and hard when it is expelled outwards beneath the peritoneum. As long as the tumour remains sessile and is not of large size, it can be left alone. If, however, it becomes pedunculated, it should be removed, because such a tumour, wandering about the peritoneal cavity, may cause peritonitis and perhaps intestinal obstruction.

### Submucous Fibroids.

*Submucous Fibroids.*—Here the menorrhagia becomes much more profuse, and eventually almost continual metrorrhagia is established. If the fibroid is of large size, one part of the tumour may be implanted in the uterine wall while the other projects into the cavity, causing great dilatation of the uterine cavity. The uterus makes efforts to expel the tumour, and thus causes pain. The patient becomes very ill from the loss of blood. The os becomes patulous and dilates, and the examining finger may pass through the cervix, or the lower pole of the tumour may bulge into the vagina. Sloughing and septic necrosis are common, and may involve part or the whole of the tumour. Under these circumstances the result may be fatal through septicæmia or peritonitis or thrombosis.

Smaller tumours may cause great hæmorrhage and may dilate the uterine cavity, and later become polypoid. Radical methods should be adopted, but here one must deal with the growth from the vagina. If hæmorrhage is profuse and the cervix patulous the operator must explore the uterus from the vagina, for if this is not done he may be tempted to perform hysterectomy when, in fact, the fibroid could easily have been shelled out of its bed per vaginam. For larger sessile tumours hysterectomy may be necessary. Polypoid tumours should always be removed per vaginam.

When a large submucous fibroid is sloughing, the necrotic portion of the tumour should first of all be removed per vaginam, followed by copious antiseptic douching. These patients are often very ill from septic absorption. If the soft necrotic portion of the tumour is cut away bit by bit until the hard normal fibroid tissue is reached, the patient's temperature will generally drop, and after some weeks the rest of the tumour and the uterus can be removed by the abdominal route.

Although one cannot help thinking that palliative treatment is often advised without due consideration of the many important points which demand attention, it must be ever a matter of regret that these very points are often neglected in advising operation. It is far better to lean toward the adoption of palliative measures than to radical operative treatment.

---

The Glasgow Maternity Hospital appeals for subscriptions to meet the cost of the additional buildings in course of erection in Ure Place. The hospital was originally started in 1834, and has occupied different sites during its history. The buildings at present drawing towards completion will meet the pressure on the present inadequate accommodation for patients; and at the same time provide a school equipped in all its departments for the teaching of practical obstetrics and gynæcology. Towards the price of the ground and the cost of the new buildings a sum of about £40,000 has been subscribed, and for the balance still required of £35,000 the directors make an earnest appeal.

